# Shape Transformations and Self-Assembly of Hairy Particles under Confinement

**DOI:** 10.3390/ijms23147919

**Published:** 2022-07-18

**Authors:** Małgorzata Borówko, Tomasz Staszewski

**Affiliations:** Department of Theoretical Chemistry, Faculty of Chemistry, Institute of Chemical Sciences, Maria Curie-Skłodowska University in Lublin, 20-031 Lublin, Poland; staszewski@umcs.pl

**Keywords:** hairy nanoparticles, slit-like pores, self-assembly, molecular dynamics

## Abstract

Molecular dynamics simulations are used to investigate the behavior of polymer-tethered nanoparticles between two inert or attractive walls. The confinement in pores creates new possibilities for controlling the shape transformation of individual hairy particles and their self-organization. We introduce a minimalistic model of the system; only chain-wall interactions are assumed to be attractive, while the others are softly repulsive. We show how the shape of isolated particles can be controlled by changing the wall separation and the strength of the interaction with the surfaces. For attractive walls, we found two types of structures, “bridges” and “mounds”. The first structures are similar to flanged spools in which the chains are connected with both walls and form bridges between them. We observed various bridges, symmetrical and asymmetrical spools, hourglasses, and pillars. The bridge-like structures can be “nano-oscillators” in which the cores jump from one wall to the other. We also study the self-assembly of a dense fluid of hairy particles in slit-like pores and analyze how the system morphology depends on interactions with the surfaces and the wall separation. The hairy particles form layers parallel to the walls. Different ordered structures, resembling two-dimensional crystalline lattices, are reported. We demonstrate that hairy particles are a versatile soft component forming a variety of structures in the slits.

## 1. Introduction

Emerging applications in various fields including photonics, electronics, sustainable energy systems, and biotechnology [1,2,3,4,5] require hybrid materials that combine the facile processing and lightweight properties of thermoplastic organic polymers with unique physical features of solid inorganic particles. Polymer-tethered inorganic cores, called “hairy” particles, naturally combine these features [6]. In the last decade, considerable academic research has been devoted to the synthesis of various hairy particles. Their properties such as the size, shape, charge, and degree of polydispersity can be reasonably well controlled during the production mechanisms to render particles with a rich variety of interactions and self-assembly properties [6].

The rapid development of hairy particle synthesis methods stimulated progress in theoretical research on their behavior in diverse systems [7]. Initially, the focus was placed on modeling the morphology of polymer canopies by changing the properties of tethers, the grafting density, the interactions of ligands with the environment, and the temperature. The first theories were based on the analogy between the hairy particles and star polymers. Ohno et al. [8] extended the mean-field theory of star polymers developed by Daoud and Cotton [9] to the polymer-tethered spherical particles of different sizes. Among theoretical approaches applied to polymer-tethered particles were also the self-consistent field model, scaling theory [10,11], and density functional theory [12,13]. Recently, ligand-tethered particles were intensively researched using fully atomistic [14,15,16,17,18,19] and coarse-grained molecular simulations [20,21]. In this way, numerous complex systems were modeled to explore different physical phenomena. For example, Grest’s group [14,15,16] dealt with spherical particles modified with various ligands immersed in water and organic solvents. However, Giri and Spohr [17,18] studied alkanethiol chain-covered gold nanoparticles in an aqueous NaCl solution and analyzed the penetration depth of water and ions into the diffuse polymer shell and its dependence on grafting density and functionalization. The hydrophobicity of monolayer protected gold nanoparticles was also explored [19]. Staszewski [20] simulated self-assembly of the ligands into “patches” on the core surface and how the type of ligands, their number, and the strength of interaction affect the particle morphology. Moreover, co-assembly of polymer-tethered particles and smaller isotropic particles was investigated [21,22]. In such mixtures, the adsorption of isotropic particles “on chains” causes the reconfiguration of the tethered chains on the core surface, leading to a change in the shape of the polymer segment cloud.

The behavior of polymer-tethered particles near solid surfaces has been examined much less frequently. The research focused mainly on the morphology of the thin films of hairy particles deposited on substrates. The structure of the surface layers depends on the ligand properties, surface chemistry, and solvent quality. It has been proven that the presence of a solid surface affects the internal structure of hairy particles. Che et al. [23] showed that, with increasing polymer-surface interaction energy, the polymer “canopy” of individual particles can spread out to increase its interaction with the surface. They studied polystyrene-grafted gold nanoparticles in a sub-monolayer regime and found strings of particles, whereas in the dense monolayer, a well-ordered hexagonal structure was observed. The wetting-dewetting stability was determined by the competition between the particle-particle and the particle-surface interactions [24]. The experimental observations were confirmed by molecular dynamics simulations [25,26,27,28,29]. As one can expect, the canopies of adsorbed hairy particles were similar to the structures formed by star polymers at the surfaces [30,31].

The mechanism of adsorption of ligand-tethered nanoparticles on solid surfaces was also investigated [25,28,32] and discussed in terms of a competition between entropic and enthalpic driving forces. Furthermore, the impact of aggregation on the adsorption of mono-tethered particles was studied. Simulations performed for mono-tethered particles showed that these particles can be adsorbed either as single particles or as different aggregates [32].

The internal morphology of hairy particles can be also controlled by confinement. Quite recently, nanoparticles modified with diblock copolymers have been studied both in a bulk systems and between two walls [33]. Each attached chain contains two parts, the first of solvophilic segments and the second of solvophobic ones. The solvophilic part of the diblock copolymer was tethered to the particle. The solvophobic segments of the copolymers were aggregated into different patches. The authors showed that the number of the patches could be determined by tuning the degree of confinement imposed on the particle.

A particularly active area of research pertains to the self-organization of the hairy particles as these particles are promising building blocks for the production of a new class of nanocomposites [3]. Therefore, much work has been devoted to understanding the mechanism of their assembly. Most of them concerned bulk systems [34,35,36,37]. However, a new strategy for controlling nanoparticle assembly is to place them in a system with a constrained geometry. The self-assembly of various nanoparticles under confinement has been already investigated, including Janus particles [38,39,40,41], heterogeneously charged particles [42], and different macromolecules [43]. Sato, Kobayashi, and Arai [44] investigated the changes in the self-assembled structure of polymer-tethered nanoparticles confined in nanotubes due to the chemical properties of polymers. Much effort has been also devoted to studying the behavior of different molecules under confinement [45,46,47].

Another possibility to steer nanoparticle self-assembly, which has attracted much attention over the past decade, is subjecting them to an external drive, such as electric or magnetic fields [48,49,50].

So far, however, there have not been systematic studies of the behavior of hairy particles between attractive surfaces. The aim of this work is to close this gap. We consider here ligand-tethered particles adsorbed in slit-like pores with inert or attractive walls.

It should be mentioned that the adsorption of hairy particles on solid surfaces is a process of industrial importance. The increasing number of various materials containing nanoparticles creates a new type of pollutant [51]. The adsorption-based methods are effective and safe procedures for their removal from water [51]. Commercial adsorbents are usually highly porous. This is also one of the reasons why we are interested in the behavior of hairy particles in the pores. In spite of the importance of this process, a theoretical approach capable of describing the adsorption of polymer-tethered particles in pores is up to now still missing.

In this work, we study an idealized coarse-grained model for polymer-tethered particles in a slit using molecular dynamics simulations. Our goals are twofold. Firstly, we show how the strength of segment-surface interactions and the wall separation affects the shapes of polymer coatings of isolated hairy particles. We analyze selected shape metrics for different sets of system parameters. We summarize our findings in the schematic diagram of the resulting structures. Secondly, we investigate the self-assembly of hairy particles adsorbed in different slits. We show what ordered structures are formed in a dense fluid of hairy particles under confinement.

The rest of the paper is organized as follows: The next section is devoted to the brief presentation of the model and simulation methodology employed. In the subsequent sections, we present and discuss our results regarding a single hairy particle and a dense fluid of hairy particles in the slits. Finally, we summarize the results and draw our conclusions.

## 2. Results and Discussion

### 2.1. A Single Hairy Particle in a Slit-like Pore

We begin our discussion by describing the behavior of a single particle with attached chains in different slits. Most of the simulations were performed for f = 30. As already mentioned, we studied slits with inert walls (repulsive segment-wall interaction) and pores with attractive walls of different strengths of segment-wall interactions, $\varepsilon_{s}^{*}\;=\;1,3,6$. We considered very narrow slits in which the potentials of both walls overlapped in the whole pore ($H^{*}\;=\;6,10,14$) and wider ones ($H^{*}\;=\;20,24,30$). For the threshold value $H^{*}\;=\;20$, the surface potentials overlapped at the plane parallel to the walls located in the pore center. However, in the middle part of the widest slits ($H^{*}\;=\;24,30$), the surface forces disappear.

**Figure 1:**
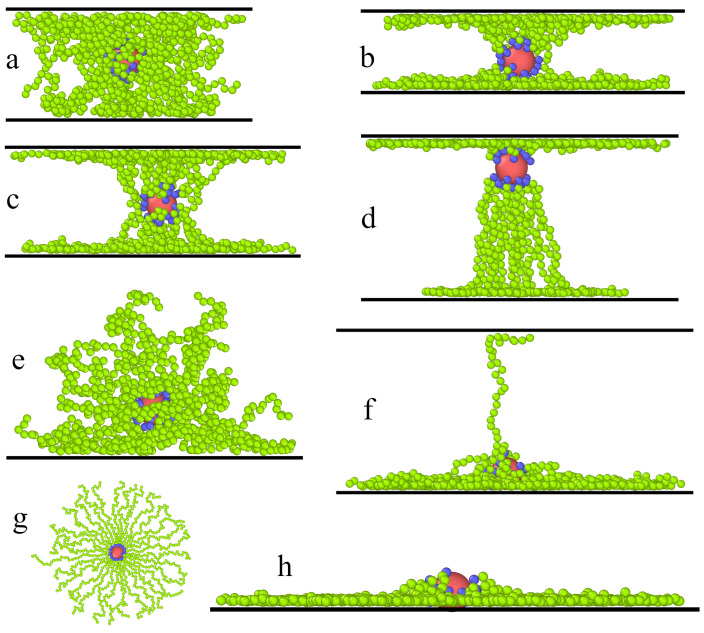
Examples of the equilibrium configurations of hairy particles in different slits. (**a**) $H^{*}\;=\;14$, $\varepsilon_{s}^{*}\;=\;1$,—pillar (P); (**b**) $H^{*}\;=\;10$, $\varepsilon_{s}^{*}\;=\;3$,— hourglass (H); (**c**) $H^{*}\;=\;14$, $\varepsilon_{s}^{*}\;=\;3$, —symmetrical spool (S); (**d**) $H^{*}\;=\;20$, $\varepsilon_{s}^{*}\;=\;6$,—asymmetrical spool (S1); (**e**) $H^{*}\;=\;24$, $\varepsilon_{s}^{*}\;=\;1$,—mound (M); (**f**) $H^{*}\;=\;20$, $\varepsilon_{s}^{*}\;=\;3$,—mound with a “plume” (M2); (**g**,**h**) $H^{*}\;=\;30$, $\varepsilon_{s}^{*}\;=\;6$,—flattened mound (M1); in (**g**), its view from above is shown. The red sphere represents the core, and blue spheres correspond to bonding segments; green spheres represent the remaining segments.

In Figure 1, we present examples of typical conformations of hairy particles in the slits. In inert slits, the segment clouds are flattened spheroids similar to those presented by Ventura Rosales et al. [33]. As the pore width increases, the flattening effect gradually weakens. For the sake of space saving, we omitted these pictures here. For slits with attractive walls, we can distinguish two basic conformations of hairy particles: bridges and mounds. In the first case, the chains are connected with both walls and, together with the core, form a bridge between them (Parts a–d). The mounds, however, are formed on one of the walls (Parts e–h). n the last case, the distance between the walls is too large for the ligands to be in an energetically profitable contact with both surfaces at the same time. Moreover, due to entropic effects, configurations with chains strongly stretched between two walls are less favorable. As a consequence, the particle “falls” onto a randomly selected wall.

**Figure 2:**
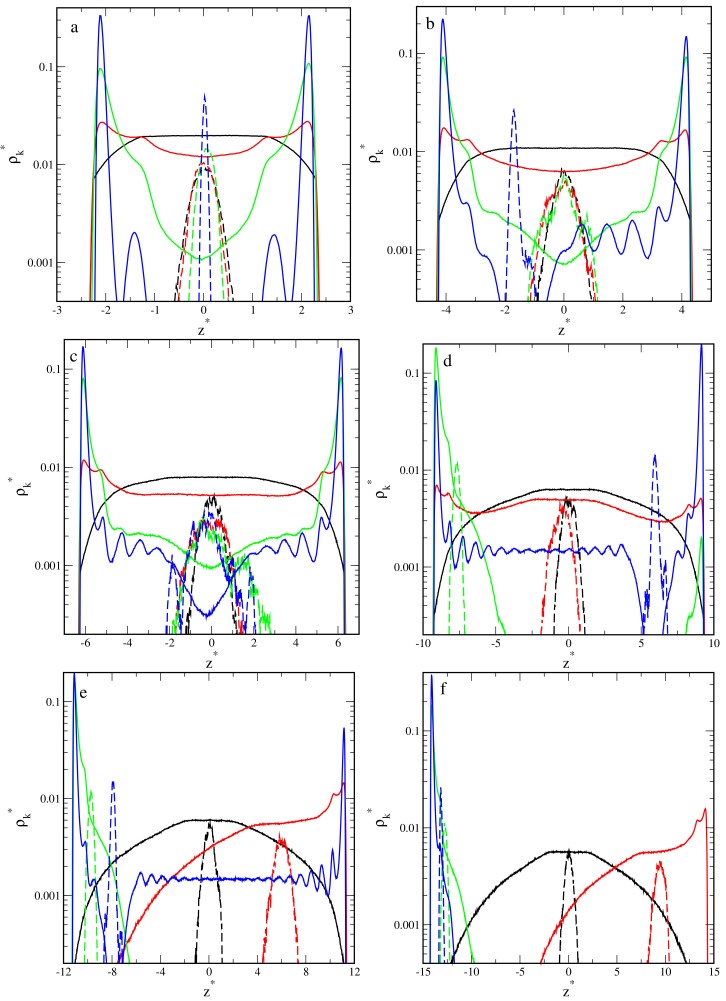
Density profiles of the chain segments (k = s, solid lines) and the cores (k = c, dashed lines) in the slits with different wall separations and different segment-wall interactions: repulsive (black) and attractive with $\varepsilon_{s}^{*}$: 1 (red), 3 (green), 6 (blue). (**a**) $H^{*}\;=\;6$, (**b**) $H^{*}\;=\;10$, (**c**) $H^{*}\;=\;14$, (**d**) $H^{*}\;=\;20$, (**e**) $H^{*}\;=\;24$, and (**f**) $H^{*}\;=\;30$. The abscissas are scaled logarithmically.

**Figure 3:**
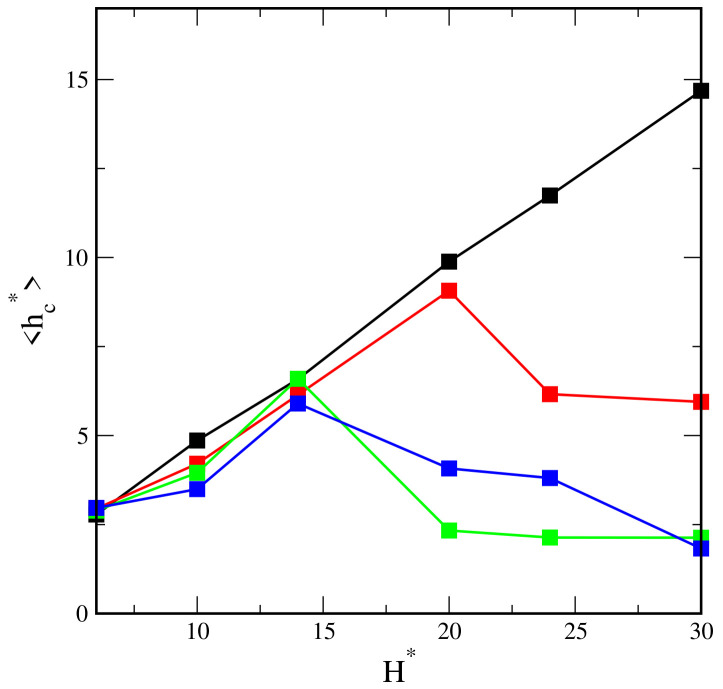
The average distance of the core from the nearest wall as a function of the wall separation for different segment-wall interactions: repulsive (black) and attractive with $\varepsilon_{s}^{*}$: 1 (red), 3 (green), 6 (blue).

We distinguished the found conformations on the basis of the density profile of segments and the center-to-wall distance of the core (see Figure 2 and Figure 3). This classification was confirmed by the observation of the equilibrium conformations and the analysis of the shape parameters. The boundaries between the above structures are not sharp and were chosen arbitrarily.

In general, the conformations classified as bridges are similar to flanged spools. We observed four spool-like shapes, namely pillars (P), symmetrical (S) and asymmetrical (S1) spools, and symmetrical (H) and asymmetrical (H1) hourglasses. The pillar density profiles had relatively low peaks at the surfaces as compared to the density at the center. In the case of the spools, the density of segments had very high and sharp peaks near the walls; moreover, the density remained relatively high in the central part of the slit. For a symmetrical spool, the core was located near the pore center, and its flanges were almost the same. On the contrary, for an asymmetrical spool, the core lied closer to one of the walls, and the flanges were different. In the case of an hourglass-like structure, however, the density of segments had a deep minimum near the core and gradually increased to reach the maxima at the walls. As previously, we distinguished symmetrical and asymmetrical hourglasses.

If the hairy particles considered here adsorb on the wall, they take the shape of a mound (M), flattened mound (M1), and the mound with a few extended chains that touch the other wall (a mound with a “plume”, M2). The structure M1 is similar to a starfish configuration of adsorbed star polymers in which full collapse of the chains onto the surface is observed [31]. In the mound, the core is detached from the wall and lies on the segment pillow. However, the core in the flattened mound almost touches the surface. The adsorbed chains spread laterally on the surface and become more stretched (see Figure 1g,h).

In Figure 2, we collected the density profiles of segments, $\rho_{s}^{*}\left(z^{*}\right)$ (solid lines), and the density profiles of cores, $\rho_{c}^{*}\left(z^{*}\right)$ (dashed lines). For each pore width, we plotted the density profiles for inert walls (black lines) and attractive walls with different energy parameters: $\varepsilon_{s}^{*}\;=\;1$ (red lines), $\varepsilon_{s}^{*}\;=\;3$ (green lines), and $\varepsilon_{s}^{*}\;=\;6$ (blue lines).

The segment density profiles help us imagine the hairy particles at confinement. One can visualize the shape of the segment cloud by rotating the density profile around the z-axis. Note that the density is presented in a logarithmic scale. We checked that all profiles in the xy-plane are symmetrical.

Now, we start to discuss the density profiles of the studied systems. Depending on their behavior, we can distinguish three groups: the pores with repulsive walls, the pores with weakly attractive walls ($\varepsilon_{s}^{*}\;=\;1$), and the pores with walls being strong adsorbents ($\varepsilon_{s}^{*}\;=\;3$ and $\varepsilon_{s}^{*}\;=\;6$).

At the first step, we analyzed the distributions of cores in the slits. In all cases, we see one Gaussian-like peak in the density profile of the core. However, the width of these peaks depends on the parameters $H^{*}$ and $\varepsilon_{s}^{*}$. A wider peak reflects stronger oscillations of the core around its average position. As has been already mentioned, the cores can be located either at the slit’s center or closer to one of the walls. In most systems, oscillations are stronger for repulsive and weakly attractive walls, while the narrow peaks are visible for highly adsorbing surfaces. However, it is interesting that for $H^{*}\;=\;14$, considerable oscillations are observed also for strongly attractive walls (see Figure 2c). We return to this phenomenon in the further part of the work.

To complete the above discussion, we show the average distance of the core from the nearest wall, $h_{c}^{*}$, in different systems (Figure 3). This reflects how much the core is pulled to the surface. In the case of inert walls, the plot $h_{c}^{*}\;vs\;H^{*}$ is a straight line with the slope 0.5, which corresponds to the location of the core at the pore center. For weakly attractive surfaces, initially, the plot is quite similar, but from $H^{*}\;=\;20$, the average distance from the wall decreases. Other relations are visible for strongly attractive walls. In this case, the cores move to one of the surfaces already in narrower slits ($H^{*}\;=\;14$).

Now, we turn to the detailed analysis of the segment density profiles. We begin with the discussion of the impact of the pore width with repulsive walls on the behavior of hairy particles. In this case, the segment density is lower at the proximity of the walls and higher near the center. As the wall separation increases, the segments accumulate in the middle part of the slit (i.e., at the plane $z^{*}\;=\;0$), and the segment cloud becomes more spherical.

Another scenario is observed for the weakly attractive walls. For narrow pores (Figure 2a–c), the segment density increases in the immediate proximity of the walls, and it remains quite high elsewhere (P). If the pore width increases, the peaks at both walls become lower, while at the middle fragment, the profile varies; for $H^{*}\;=\;6,10$, we see here a shallow minimum; for $H^{*}\;=\;14$, there is a plateau; a low wide maximum is seen for $H^{*}\;=\;20$. After reaching a certain threshold value, the particle “falls” on one wall ($H^{*}\;=\;24$ and $H^{*}\;=\;30$). Obviously, the core is located close to this wall. This is reflected in the density profiles; the segment density reaches a maximum near the surface and slowly decreases with increasing distance from this wall (M).

We end our discussion with the analysis of the results obtained for slits with very attractive walls. Firstly, let us focus on narrow slits (Figure 2a–c). We see here that the segment profiles have very high and sharp peaks at surfaces and gradually decrease to deep minima at $z^{*}\;=\;0$. We classified these conformations as hourglasses. In this case of the narrowest slit, two well-pronounced peaks at the surfaces are visible, while the density elsewhere is extremely low. For $H^{*}\;=\;10$ and $H^{*}\;=\;14$, there is a deep minimum in the segment density profiles, and the cores lie closer to one of the walls (H1).

A different picture of hairy particle transformations is observed in wider pores (Figure 2d–f). A non-intuitive effect of segment-wall interactions is found for the pore with $H^{*}\;=\;20$ (Figure 2d). In the case of $\varepsilon_{s}^{*}\;=\;3$, the segments accumulate mainly at one of the walls. However, for the stronger interactions ($\varepsilon_{s}^{*}\;=\;6$), the peaks at the walls have comparable heights. The cores lie near the walls covered by more segments. This results from a longer range of the potential (5) for $\varepsilon_{s}^{*}\;=\;6$. Moreover, if $\varepsilon_{s}^{*}\;=\;3$, the density of segments decreases to an extremely low value at the pore center. We monitored the system configurations and concluded that the particle is adsorbed on a wall, but a few chains can still be held by the other one (M2). However, for $\varepsilon_{s}^{*}\;=\;6$, asymmetrical spools are formed (S1).

As the pore width rises to $H^{*}\;=\;24$ (Figure 2e), for $\varepsilon_{s}^{*}\;=\;3$, the particle falls on a random surface (M), but for $\varepsilon_{s}^{*}\;=\;6$, still, a bridge is formed. In the case of the widest pore (Figure 2f), the hairy particle always adsorbs on one of the walls. For stronger adsorbents, the cloud of segments is more flattened, as evidenced by a high and sharp peak near the surface.

It should be emphasized that the hourglass and spool conformations have not been reported in the literature so far.

The size and shape of the hairy particle can be described by means of various parameters [52]. Firstly, we calculated the radius of gyration of the cloud of segments:

(1) $$R_{g}^{2}\;=\;\frac{1}{N^{\prime }}\langle \sum_{i=1}^{N^{\prime }}r_{i0}^{2}\rangle$$

where $r_{i0}\;=\;r_{i}\;-\;r_{0}$, $r_{i}$ and $r_{0}$ are the positions of the ith segment and the center of mass, respectively, and $N^{\prime }\;=\;fM$.

**Figure 4:**
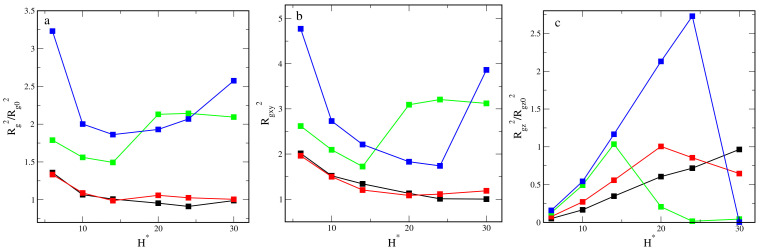
The squared radius of gyrations (**a**), the averages of the components of the squared radius of gyration in directions parallel to the walls (**b**), and the *z*-th component of the squared radius of gyration (**c**) plotted as functions of the wall separation for different segment-wall interactions: repulsive (black) and attractive with $\varepsilon_{s}^{*}$: 1 (red), 3 (green), 6 (blue). All the radii of gyration are divided by their bulk counterparts.

We resolved the vectors $r_{i0}$ in Equation (6) into components parallel to the axes *x*, *y*, *z* and calculated the corresponding radii of gyration labeled $R_{g\alpha }^{2}$ (α = x,y,z), the sum of which equals $R_{g}^{2}$. The results are depicted in Figure 4. All the radii of gyration are divided by their bulk counterparts. We carried out a simulation for bulk systems and found $R_{g0}\;=\;9.16$. In bulk systems, always $R_{gx0}\;=\;R_{gy0}\;=\;R_{gz0}$. We can define the degree of confinement for the hairy particle as $d^{\prime }\;=\;H^{*}/\left(2R_{g0}\right)$. Thus, we considered the degrees of confinement ranging from 0.327 ($H^{*}\;=\;6$) to 1.634 ($H^{*}\;=\;30$).

For repulsive and weakly attractive walls, as the slit becomes wider, the total radius of gyration slowly decreases to the bulk values (see Figure 4a). As expected, for the inert walls, the ratio $R_{g}^{2}/R_{g0}^{2}$ decreases to 1. In the case of strongly attractive walls, the total radii of gyration are several times higher, and the curves $R_{g}^{2}\;vs\;H^{*}$ are completely different. Initially, they decrease to minima at $H^{*}\;=\;14$. Then, the radius of gyration gradually increases for $\varepsilon_{s}^{*}\;=\;6$, but for $\varepsilon_{s}^{*}\;=\;3$, it achieves a plateau at $H^{*}\;=\;20$. An increase in the parameter $\varepsilon_{s}^{*}$ causes the increase of $R_{g}^{2}$ for narrow pores, but the opposite effect is observed for the widest ones. In Figure 4b, we plot the average of the components of the radius of gyration in directions parallel to the walls, $R_{gxy}^{2}\;=\;0.5\left(R_{gx}^{2}/R_{gx0}^{2}\;+\;R_{y}^{2}/R_{gy0}^{2}\right)$, as functions of the wall separation. The curves $R_{gxy}^{2}$ vs. $H^{*}$ are very similar to those from Part a. This means that the contribution of components parallel to the walls to the total radius of gyration is dominating. However, the component $R_{gz}^{2}$ varies with the pore width in a completely different way (Figure 4c). For the inert walls, $R_{gz}^{2}/R_{gz0}^{2}$ gradually increases from a very low value to unity. In other cases, these curves have one maximum whose position depends on the segment-wall interactions. For $\varepsilon_{s}^{*}\;=\;1$, the maximum appears at $H^{*}\;=\;20$, while for $\varepsilon_{s}^{*}\;=\;6$, it is at $H^{*}\;=\;24$. At these points, the transformation to the M-structures is observed. However, for the intermediate value $\varepsilon_{s}^{*}\;=\;3$, a decrease in $R_{gz}^{2}$ begins in the narrow pore with $H^{*}\;=\;14$, where the asymmetrical spools are formed.

Next, we used the shape parameters defined by means of the gyration tensor [52]:

(2) $$G_{\alpha \beta }\;=\;\frac{1}{N^{\prime }}\langle \sum_{i=1}^{N^{\prime }}\left(r_{i,\alpha }-r_{0,\alpha }\right)\left(r_{i,\beta }\;-\;r_{0,\beta }\right)\rangle$$

where $r_{i,\alpha }$ and $r_{0,\alpha }$ are the α component (α,β = x,y,z) of the i-th segment and of the center of mass of the segment cloud, respectively.

Diagonalization of the gyration tensor yields its eigenvalues $\lambda_{i}$ (i = 1,2,3), which we order as $\lambda_{1}\;\geq \;\lambda_{2}\;\geq \;\lambda_{3}$. The eigenvalues are proportional to the principal half-axes of an equivalent ellipsoid. Then, three invariants can be obtained $I_{1}\;=\;\lambda_{1}\;+\;\lambda_{2}\;+\;\lambda_{3}$, $I_{2}\;=\;\lambda_{1}\lambda_{2}\;+\;\lambda_{1}\lambda_{3}\;+\;\lambda_{2}\lambda_{3}$, and $I_{3}\;=\;\lambda_{1}\lambda_{3}\lambda_{3}$. These values can be used to define shape descriptors of the segment cloud, such as the relative shape anisotropy and the prolateness [33,52]. This procedure provides also an alternative method of calculating the radius of gyration because $R_{g}^{2}\;=\;I_{1}$ [52].

The relative shape anisotropy is defined as

(3) $$\kappa \;=\;1\;-\;3\langle I_{2}/I_{1}^{2}\rangle ,$$

and takes values between 0 and 1, with the minimum value describing an object with perfect spherical symmetry and the maximum value corresponding to rigid rods. However, for a regular planar array, κ = 0.25 [52].

The prolateness is given by

(4) $$S\;=\;\langle \left(3\lambda_{1}\;-\;I_{1}\right)\left(3\lambda_{2}\;-\;I_{1}\right)\left(3\lambda_{3}\;-\;I_{1}\right)/I_{1}^{3}\rangle$$

with its value ranging from −0.25 to 2, where the negative values indicate oblate shapes, whereas the positive ones correspond to prolate objects. For perfectly oblate clouds $\lambda_{1}\;=\;\lambda_{2}\;>\;\lambda_{3}$, while in perfectly prolate ones, the shorter axis of the ellipsoid are the same ($\lambda_{1}\;>\;\lambda_{2}\;=\;\lambda_{3}$) [53].

**Figure 5:**
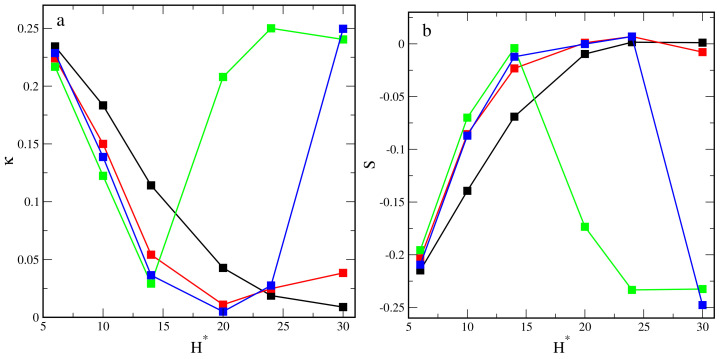
The relative shape anisotropy (**a**) and the prolateness (**b**) plotted as functions of the wall separation for different segment-wall interactions: repulsive (black) and attractive with $\varepsilon_{s}^{*}$: 1 (red), 3 (green), 6 (blue).

The shape parameters of the considered hairy particles in the different slits are shown in Figure 5. We begin with the presentation of how these parameters depend on the wall separation in the slits with inert walls. One can see that the relative shape anisotropy gradually decreases from 0.23 to a value close to zero, corresponding to spherical objects. On the contrary, the prolateness increased from −0.22 to 0, indicating the transformation from oblate structures under the strong confinement to spherical ones in the wide pores. For attractive walls, the shape of hairy particles varies in a more complicated way. Note that the relative shape anisotropy is very low, which suggests the existence of spherical-like clouds. As the pore width rises, κ decreases to a minimum and increases again. For $\varepsilon_{s}\;=\;1$ and $\varepsilon_{s}\;=\;6$, the minima are at $H^{*}\;=\;20$, while for $\varepsilon_{s}\;=\;3$, they are at $H^{*}\;=\;14$. Then, the relative shape anisotropy increases slightly for the “weak” walls and rises rapidly for the “strong” walls. In the latter case, the flattened mounds are formed on the surface of the widest pores. The prolateness increases for the weakly attractive walls, while for the strong attraction, a sharp decline is visible after the initial rise. This is associated with the formation of flat structures at the surface.

The changes in the shape parameters reflect the transformations of the hairy particles associated with an increase in the pore width. However, the confinement between the attractive walls causes the formation of unique structures, which differ significantly from the model ellipsoids. As a consequence, these parameters cannot describe their geometry with all details. Nevertheless, the used shape metrics well characterize the observed particle conformations. The analysis of the shape parameters provides information consistent with our conclusions resulting from the discussion of the components of the radius of gyration.

**Figure 6:**
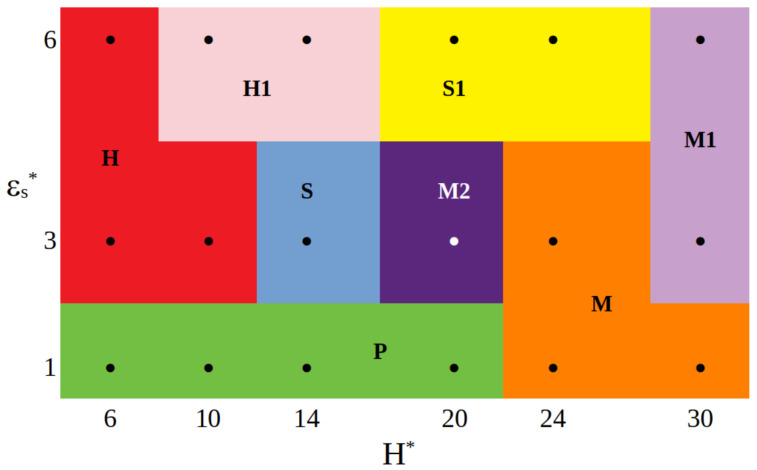
The sketch diagrams of the particle structures in the coordinates $\varepsilon_{s}^{*}$ vs. $H^{*}$ for a hairy particle. Black circles correspond to simulation points. The boundaries between structures are arbitrarily chosen.

In Figure 6, we present the overview of the particle conformations observed for different combinations of the strength of attractive segment-wall interactions and the wall separation. The boundaries between the structures are introduced arbitrarily. For weakly attractive surfaces, the pillars are observed in narrower pores, while for the wide slits ($H_{s}^{*}\;=\;24$ and $H_{s}^{*}\;=\;30$), particles fall on one of the walls forming mounds (M). In the case of $\varepsilon_{s}^{*}\;=\;3$, as the wall separation increases, the particle transforms from a symmetrical hourglass (H = 6,10), through a symmetrical spool (H = 14) and a mound with “plume” ($H^{*}\;=\;20$), to a flattened mound ($H^{*}\;=\;30$). However, for the most strongly adsorbing walls, we see the structures H (H = 6), H1 (H = 10,14), S1 (H = 20,24), and M1 (H = 30) in sequence. We see that the shape of the hairy particle depends on both the wall separation and the strength of segment-wall attraction.

**Figure 7:**
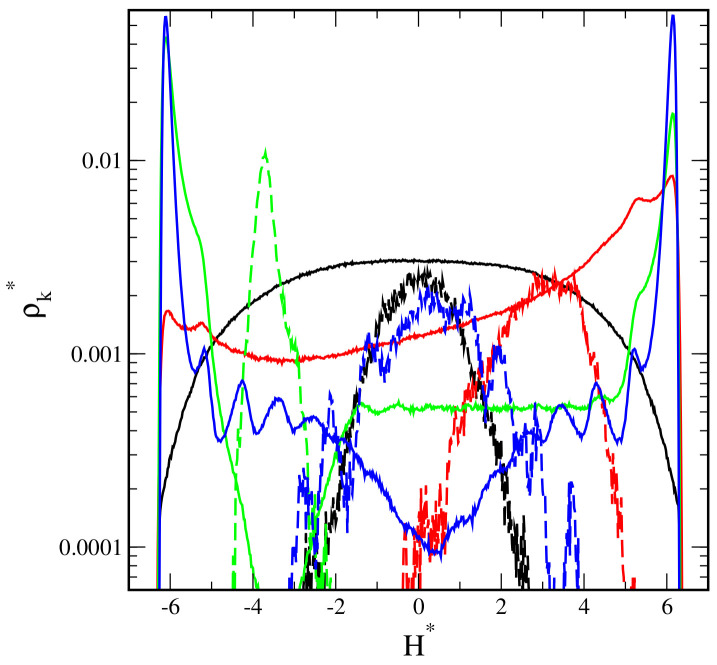
Density profiles of the chain segments (k = s, solid lines) and the cores (k = c, dashed lines) for f = 10, the wall separation $H^{*}\;=\;14$, and different segment-wall interactions: repulsive (black) and attractive with $\varepsilon_{s}^{*}$: 1 (red), 3 (green), 6 (blue). The abscissa is scaled logarithmically.

Interesting effects were reported for systems involving hairy particles with very low grafting density [36]. We supplemented our research with calculations for particles with only f = 10 chains attached. As one can expect, in bulk systems, these particles are smaller than those with f = 30. The radii of gyration are $R_{g0}\;=\;7.73$. We carried out the simulations for slits with the wall separation $H^{*}\;=\;14$. Figure 7 shows the obtained density profiles. Let us compare them with those obtained for f = 30 (Figure 2c). Of course, now, the density of segments is much less. Moreover, for weakly attractive walls, the profile has a maximum much higher at one of the walls. The asymmetrical pillars are formed, which is not observed in the previous case. Furthermore, for $\varepsilon_{s}^{*}\;=\;3$, a decrease of the number of ligands causes a change of the structure (from S to S1). However, for $\varepsilon_{s}^{*}\;=\;6$, in both cases, H1 conformations are found.

**Figure 8:**
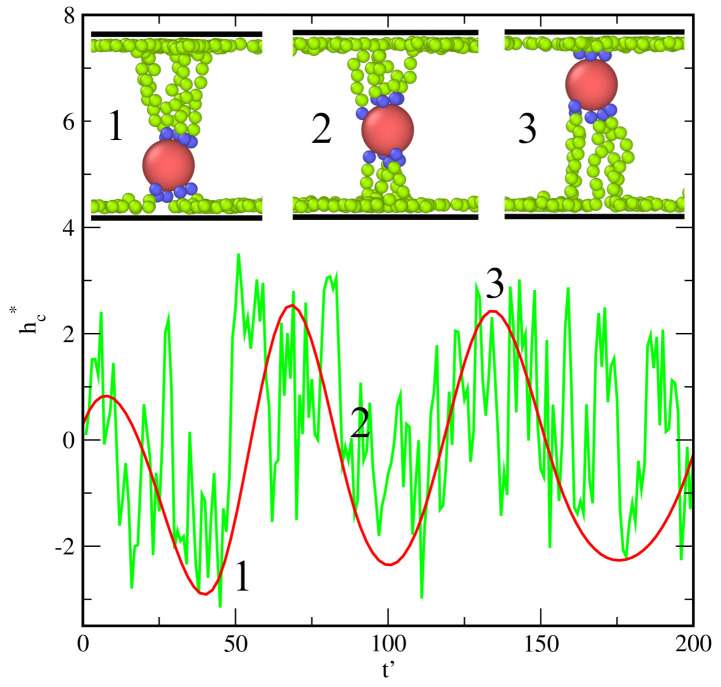
The time evolution of the distance of the core from a plane parallel to the walls, located at $H^{*}\;=\;0.5$, for $H^{*}\;=\;14$ and $\varepsilon_{s}^{*}\;=\;6$. In the top row, examples of representative structures are shown.

However, the most important finding to point out regarding the particles with a low grafting density is that we observe here much stronger oscillations of cores around their average positions. In Figure 8, an example of the time evolution of the distance of the core from the nearest surface is shown (lower panel) and the corresponding conformations (top panel). Due to the competition between the attraction by both walls, the distance of the core from the pore center changes (green line). These changes are significant. The function $h_{c}^{*}\left(t^{\prime }\right)$ resembles the chaotic noise line. However, we found here a certain regularity and state that this distance changes periodically (red line). We see that the core approaches one wall, then the other. An exemplary configuration with the core close to the bottom surface is assigned as “1” and the configuration with the core in the center of the pore as “2”, and the configuration “3” corresponds to the core near the top wall. The numbers in the lower panel show when these configurations appear in the system. We conclude that the particle behaves like a kind of “a nano-oscillator”, in which the core jumps from one wall to the other. A similar effect is also observed for particles with f = 30, but is less pronounced (Figure 2b,c).

### 2.2. A Fluid Involving Hairy Particles in Slit-like Pores

**Figure 9:**
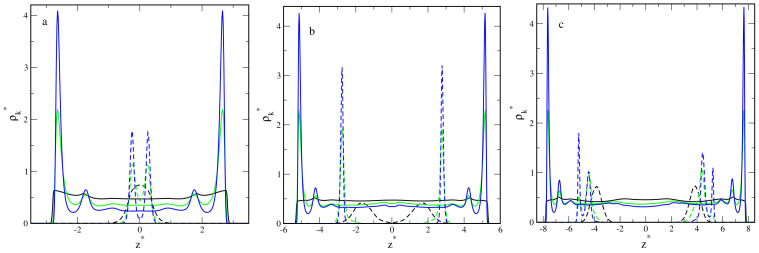
Density profiles of the chain segments (k = s, solid lines) and the cores (k = c, dashed lines) in the slits with different wall separations and different segment-wall interactions: repulsive (black) and attractive with $\varepsilon_{s}^{*}$: 3 (green), 6 (blue). (**a**) $H^{*}\;=\;7$, (**b**) $H^{*}\;=\;12$, and (**c**) $H^{*}\;=\;17$.

We carried out a simulation for a fluid involving many hairy nanoparticles confined between two walls. In order to reduce the computation time, we performed them for shorter ligands, assuming that M = 10 and f = 30. We considered only narrow slits with $H^{*}\;=\;7,12,17$, where the potentials of both walls overlap. First, we performed simulations for single particles. The obtained shapes of individual hairy particles were analogous to those reported for longer tethers. Next, we studied the structure of relatively dense systems in which the density of nanoparticles $\rho_{0}^{*}\;=\;0.5$. Figure 9 presents the density profiles of cores and segments for repulsive and attractive segment-wall interactions. We see here that in the narrowest pore (part a), the cores form one “levitating” monolayer in the pore center. This results from the superposition of force fields generated by two walls. In the case of repulsive segment-wall interactions, the core density has one low and wide peak, while the segments are almost evenly distributed throughout the slit. For attractive interactions, a single peak in the core density profiles partially splits into two high and narrow peaks. Due to geometrical reasons and the attraction by both walls, the cores form a slightly corrugated layer in the middle of the pore. The segments, however, accumulate at the surfaces, two well-pronounced layers near each surface are clearly visible. The layer of cores lies on the pillows built of segments. In the wider slits (Parts b, c), we observed two layers of particles located closer to the walls. For attractive segment-wall interactions and $H^{*}\;=\;12$, the core profiles have two single, narrow peaks, which indicates the formation of “flat” layers. However, for $H^{*}\;=\;17$, the double peaks corresponding to corrugated layers are observed.

It is interesting to analyze the distribution of cores in the slits and within these monolayers. In order to analyze the ordering in the monolayers, we used the global 2D bond-orientational order parameter, $Q_{k}^{2D}$, defined as [54]

(5) $$Q_{k}^{2D}\;=\;\frac{1}{N_{bond}}|\sum_{i}\sum_{j\neq i}\exp\left(ki\phi_{ij}\right)|$$

where *i* runs over all cores of the system, *j* runs over all neighbors of *i*, $\phi_{ij}$ denotes the angle between the bond connecting cores *i* and *j* and an arbitrary, but fixed reference axis, $N_{bond}$ is the number of bonds in the system, while k = 2,3,4,5,6. We assumed that two particles are neighbors if their distance is less than the location of the first minimum in the pair correlation function (radial distribution function). The pair correlation function describes the probability of finding the center of a particle at a given distance from the center of a reference particle. The first maximum in the core-core correlation function corresponds to the cores that are the nearest neighbors of a given core.

We estimated the parameters $Q_{k}^{2D}$ for layers parallel to the walls. We assigned to the given layer all cores whose coordinates $z^{*}$ range from the assumed values $z_{l}^{*}$ to $z_{u}^{*}$. We assumed that $z_{l}^{*}$ and $z_{u}^{*}$ correspond to the position of the first minimum before and after the peak in the core density, respectively. In the same way, we calculated the parameters $Q_{k,p}^{2D}$ for cores in the bottom and upper layers projected onto one plane. This allowed us to check the existence of correlations between these layers.

**Figure 10:**
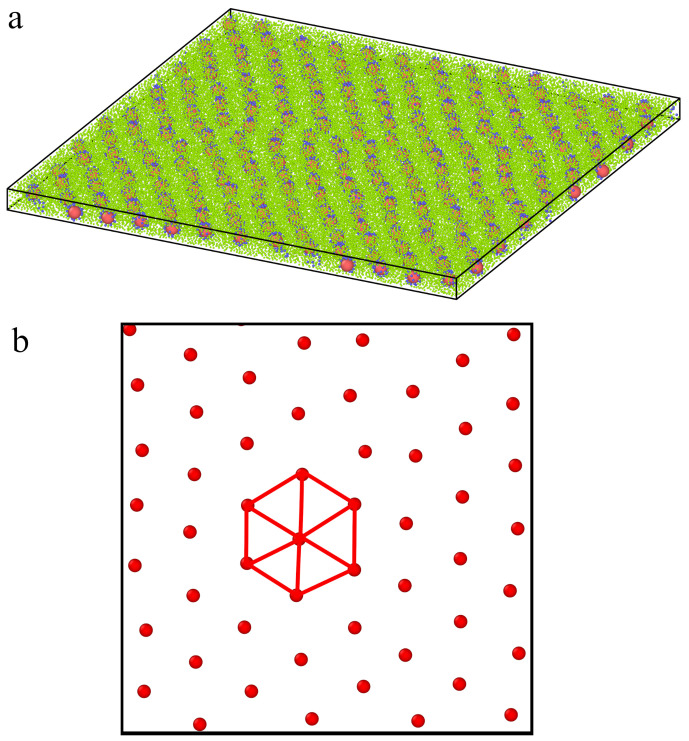
Structure of a fluid confined in the narrowest slit with attractive walls, $H^{*}\;=\;7$ and $\varepsilon_{s}^{*}\;=\;6$. (**a**) The configuration of the system, (**b**) the distribution of the cores (view from above).

Let us discuss the behavior of systems with the repulsive segment-wall interactions and attractive segment-wall interactions for $\varepsilon^{*}\;=\;6$. In the narrowest slits, the cores form a highly ordered hexagonal lattice: $Q_{6}^{2D}\;=\;0.859$ (repulsive walls) and $Q_{6}^{2D}\;=\;0.842$ (attractive walls). The results for attractive walls are presented in Figure 10. In Part b, we show the distribution of the cores in a fragment of the system. Indeed, the cores are arranged on a hexagonal lattice.

**Figure 11:**
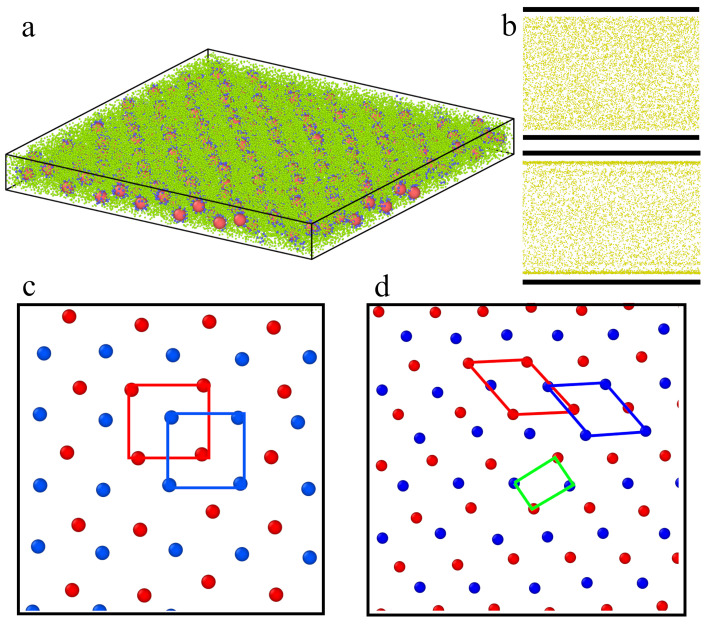
Structure of a fluid confined in the slit of width $H^{*}\;=\;12$—(**a**) configuration of the system with repulsive walls ($\varepsilon_{s}^{*}\;=\;6$), (**b**) the distribution of segments for inert walls (top panel) and attractive walls (bottom panel), (**c**,**d**) the cores projected on one plane parallel to the walls for inert surfaces (**c**) and attractive surfaces. Red points correspond to the top layer; blue points are for the bottom layer.

Interesting effects are observed in the slit of width $H^{*}\;=\;12$ (see Figure 11). In Part a, an exemplary configuration of the system is presented, while in Part b, we show the distribution of segments in the pore for repulsive (top panel) and attractive walls (bottom panel). The accumulation of segments near attractive surfaces is clearly visible. Structures formed by the cores are presented in Parts c (repulsive walls) and d (attractive walls). We show here the cores from bottom (red) and top (blue) layers projected onto one plane. In the case of repulsive segment-wall interactions, the cores are arranged on square lattices, and the average value of the bond-orientational order parameter calculated for two layers is $Q_{4,av}^{2D}\;=\;0.913$. However, this parameter calculated for all cores projected on one plane equals $Q_{4,p}^{2D}\;=\;0.799$. One can conclude that the layers are highly correlated: one is shifted relative to the other, as we show in Figure 11c. However, a different morphology has layers formed near highly attractive walls, where parallelogram lattices are found. In this case, $Q_{6,av}^{2D}\;=\;0.717$ and $Q_{6,p}^{2D}\;=\;0.323$, while $Q_{4,av}^{2D}\;=\;0.166$ and $Q_{4,p}^{2D}\;=\;0.704$. We see that the cores projected on one plane form an almost rectangular lattice.

In the widest slits considered here, uncorrelated hexagonal layers are formed for repulsive, as well as for attractive walls. We obtained the following values of the bond-orientational order parameters: $Q_{6,av}^{2D}\;=\;0.755$, $Q_{6,p}^{2D}\;=\;0.270$, $Q_{4,p}^{2D}\;=\;0.358$ (repulsive walls) and $Q_{6,av}^{2D}\;=\;0.811$, $Q_{6,p}^{2D}\;=\;0.273$, $Q_{4,p}^{2D}\;=\;0.294$ (attractive walls).

In summary, our simulations clearly demonstrate that densely packed hairy particles can form ordered structures between two walls. The system morphology depends on the interactions with the surfaces and the wall separation.

### 2.3. A Comparison of the Results with Previous Studies

As already mentioned, relatively little research has focused on the behavior of hairy particles in the pores. However, more attention has been given to the adsorption of nanoparticles on flat surfaces. Depositing hair particles on a substrate changes the grafted layer structure and can allow them to form highly ordered arrays. The structure of adsorbed film depends on the properties of polymer-grafted nanoparticles, surface chemistry, the quality of the solvent, and the deposition process used.

Experimental and theoretical studies [23,24,25,26,27,28,29] proved that the presence of a solid surface affects the structure of a single hairy particle, as well as the morphology of monolayers deposited on substrates. Among the most important parameters that influence the structure of hairy particles near surfaces are the number of tethered chains, their lengths, and the strength of interactions with the substrate.

The experiments performed by Che et al. [23] showed that a single hairy particle at the attractive surface becomes the shape of a “mound” of segments. They proved that, with increasing polymer-surface interaction energy, the polymer “canopy” of an individual hairy nanoparticle spreads out to increase its interaction with the surface. The particle configuration becomes more and more flattened. These observations have been also confirmed by molecular simulations carried out for polymer-tethered particles [25,26,29] and star polymers [31].

In this work, we studied the behavior of hairy particles in slits of different widths. For sufficiently wide pores, adsorption on individual surfaces proceeds independently. In this case, we obtained results qualitatively consistent with those previously published. However, we discussed the configuration of a polymer coating near a substrate with all details using the shape parameters. We showed how the strength of interactions with the surface influences the shape characteristics.

In narrow pores, however, hairy particles form completely different structures. Ventura Rosales et al. [33] studied the formation of patches in a single particle modified with diblock copolymer brushes in the bulk and between two inert walls. Upon increasing the confinement, the brush progressively deformed, and the number of patches changed in a non-trivial fashion. They analyzed the shape of segment clouds in pores with non-attractive walls. In this case, the polymer canopies always resembled flattened ellipsoids. For the repulsive walls, we obtained the same results.

Our most important findings, however, are associated with the attractive walls. For such slits, we observed new structures that were not reported in the literature. Under certain conditions, the particle forms a bridge between the walls. We discussed the impact of the wall separation and segment-wall interactions on the shape of a single particle.

In Section 2.2, we discussed the morphology of a dense fluid built of hairy particles in different slits. We obtained the structures of different degrees of ordering. Unfortunately, there are no experimental data for such systems. However, numerous investigations show that the solid surface influences the morphology of films built of hairy nanoparticles on substrates [23,24,27,29]. In the sub-monolayer region, the surface films investigated by Che et al. [23] contained strings of particle agglomerates, whereas the monolayers consisted of well-ordered hexagonal lattices. Either and Hall [27] studied ultrathin films containing polymer-grafted nanoparticles using molecular dynamics. They observed regular hexagonal two-dimensional structures with different spacing of the particles depending on the grafting density. In our previous work, we found the same structure [29].

When hairy particles are confined between two walls, we observed the formation of layers parallel to the surfaces. In very narrow pores, the cores form one “levitating” monolayer in the pore center. In wider pores, we found two layers located near the walls. In these slabs, the cores are arranged on different lattices (hexagonal, square, parallelogram) depending on the segment-wall interactions. Similar structures, namely “levitating slabs” and multilayers, were also found in slits with adsorbed Janus particles [40,41].

Recently, the effect of the chemical design of grafted polymers on the self-assembled morphology of polymer-tethered nanoparticles in nanotubes has been investigated [44]. Three types of tethered polymer NP models were examined: homo hydrophilic, diblock hydrophilic-hydrophobic, and diblock hydrophobic-hydrophilic. These results cannot be directly compared with our simulations because of the different symmetry of the pores.

The understanding of the self-assembly of hairy nanoparticles under different conditions is very important for modeling novel systems that may find applications in nano-optical devices or for nanopatterning.

## 3. Model and Simulation Methodology

We considered polymer-tethered particles between two parallel walls. A single particle consists of a spherical core of diameter $\sigma_{c}$ with attached *f* linear arms. Each arm contains *M* tangentially jointed spherical segments of identical diameters, $\sigma_{s}\;=\;\sigma$. The chains are perfectly flexible. We used an implicit solvent model, that is no solvent molecules are present in the system, but the interactions should be treated as effective, solvent-mediated ones.

Bonded neighbors along the backbone of a chain are held together by the harmonic segment-segment potentials:

(6) $$u^{\left(b\right)}\;=\;k{\left(r-d\right)}^{2},$$

where *r* is the distance between segments and $d\;=\;\sigma_{s}$.

We assumed that the first monomer of each ligand is tethered to the core via the harmonic potential as in (1) with $d\;=\;\sigma_{cs}$ and $k\;=\;k_{cs}$. In this case, ligands can slide over the core; in other words, they are mobile [36].

The interactions between all “atoms” (cores and segments) are modeled through the shifted-force Lennard-Jones potential [55]:

(7) $$u^{\left(ij\right)}\;=\;\left\{\begin{array}{cc} 4\varepsilon_{ij}\left[{\left(\sigma_{ij}/r\right)}^{12}\;-\;{\left(\sigma_{ij}/r\right)}^{6}\;\right]\;+\;\Delta u^{\left(ij\right)}\left(r\right), & \;\;\;r\;<\;r_{cut}^{\left(ij\right)},\\ 0, & \;\;\;\mathrm{otherwise}, \end{array}\right.$$

where

(8) $$\Delta u^{\left(ij\right)}\left(r\right)\;=\;-\left(r-r_{cut}^{\left(ij\right)}\right)\partial u^{\left(ij\right)}\left(r_{cut}^{\left(ij\right)}\right)/\partial r,$$

In the above, $r_{cut}^{\left(ij\right)}$ is the cutoff distance, $\sigma_{ij}\;=\;0.5\left(\sigma_{i}\;+\;\sigma_{j}\right)$ (i,j = c,s), while $\varepsilon_{ij}$ characterizes the strength of interactions between species *i* and *j*. To switch on or switch off attractive interactions, one can use the cutoff distance. For repulsive interactions, we assume that $r_{cut}^{\left(ij\right)}\;=\;\sigma_{ij}$.

The energy of the interactions of the segments and core with both surfaces is the following sum:

(9) $$v_{k}\left(z^{\prime }\right)\;=\;v^{\left(1\right)}\left(z^{\prime }\right)\;+\;v^{\left(2\right)}\left(H\;-\;z^{\prime }\right),$$

while $z^{\prime }$ is the distance from the surface labeled as “1” and $v^{\left(i\right)}$, (i = 1,2) describes interactions with the *i*-th wall, which are modeled by the 9-3 Lennard-Jones equation:

(10) $$v_{k}\left(z^{\prime }\right)\;=\;\left\{\begin{array}{cc} \frac{2}{15}\varepsilon_{s}^{\left(k\right)}\left[{\left(\sigma_{k}/z^{\prime }\right)}^{9}\;-\;{\left(\sigma_{k}/z^{\prime }\right)}^{3}\;\right], & \;\;\;z^{\prime }\;<\;z_{cut}^{\left(k\right)},\\ 0, & \;\;\;\mathrm{otherwise}, \end{array}\right.$$

where $z_{cut}^{\left(k\right)}$ is the cutoff distance, while $\varepsilon_{s}^{\left(k\right)}$ is the parameter characterizing the interactions of the *k*-th component with a wall (k = c,s). As previously, to switch on or switch off attractive interactions with the walls, we use the cutoff distance parameters. In the case of attractive segment-wall interactions, $z_{cut}^{\left(k\right)}\;=\;10\sigma$, while for repulsive interactions, we use the cutoff distance parameter such that $v_{k}\left(z_{cut}^{\left(k\right)}\right)\;=\;0$. The energy of the wall potential is shifted so that it falls to zero at the cutoff distance.

We introduce the standard units. The diameter of segments is the distance unit, $\sigma \;=\;\sigma_{s}$ the segment-segment energy parameter, and $\varepsilon \;=\;\varepsilon_{ss}$ the energy unit, and the mass of a single segment is the mass unity, $m\;=\;m_{s}$. The basic unit of time is $\tau \;=\;\sigma \sqrt{\varepsilon /m}$. We assume that the gravity effects are negligible. The energy constants of the binding potentials (1), $k\;=\;k_{cs}$, are $1000\;\varepsilon /\sigma^{2}$.

The behavior of the system depends on the strengths of interactions between all single entities: cores and segments, as well as their interactions with the substrate. Various combinations of interaction parameters were assumed in the simulations of hairy particles in bulk systems. These models are summarized in [29,36]. Furthermore, different interactions with the surface can be considered: (i) all repulsive interactions, (ii) all attractive interactions, (iii) attractive core-substrate interactions and repulsive segment-substrate interactions, and inversely, (iv) attractive segment-substrate interactions and attractive segment-substrate interactions.

In order to limit the number of parameters, in this paper, we only considered a very simple model. We assumed repulsive interactions between particles (core-segment, segment-segment interactions). Similarly, core-substrate interactions are repulsive. On the contrary, chains can be both repelled and attracted by the walls (repulsive or attractive segment-wall interactions). Thus, we considered the good solvent conditions. The ligands are solvophilic, while the walls can be inert or solvophobic. Such a simple model allows us to show a “pure” impact of walls on the morphology of polymer-tethered particles under confinement that disturbs the spherical symmetry of the cloud of segments. We assumed that both walls are identical and studied slits with different widths.

We used the reduced distances $l^{*}\;=\;l/\sigma$, the reduced energies $E^{*}\;=\;E/\varepsilon$, and the reduced temperature $T^{*}\;=\;k_{B}T/\varepsilon$, where $k_{B}$ is the Boltzmann constant. We define also the reduced densities: $\rho_{k}^{*}\;=\;\rho_{k}\sigma^{3}$ (k = c,s), where $\rho_{c}\;=\;N_{c}/V$ is the number density of cores, $\rho_{s}\;=\;N_{c}fM/V$ is the density of segments, $N_{c}$ denotes the number of particles (cores), and *V* is the volume of the system. The total reduced density of the system is expressed as $\rho^{*}\;=\;\rho_{c}^{*}\;+\;\rho_{s}^{*}$. Later in the work, we use the simpler symbol for the energy parameter $\varepsilon_{s}^{*}\;=\;\varepsilon_{s}^{*(s)}$.

We performed simulations for $\sigma_{c}^{*}\;=\;4$. We focused only on equilibrium structures of particles, so the mass of the core was arbitrarily set to $m_{c}^{*}\;=\;4$. In a coarse-grained model of polymers, the segments can represent several molecular fragments. Depending on the conditions, each bead can comprise 1–3 (or even more) such groups. We assumed that M = 30 or M = 10, while f = 30, and several simulations were carried out for f = 10. Moreover, $\varepsilon_{ij}^{*}\;=\;1$. We studied slits with inert walls (repulsive segment-wall interaction) and pores with attractive walls of different strengths of segment-wall interactions, $\varepsilon_{s}^{*}\;=\;1,3,6$. We considered several wall separations ranging from $H^{*}\;=\;6$ to $H^{*}\;=\;30$. Similar values of the parameters were assumed in the previous simulations of polymer-tethered particles [21,22,28,29,35].

We performed molecular dynamics simulations using the LAMMPS package [56,57], followed by post-processing by means of in-house codes to evaluate the system observables. A Nose-Hoover thermostat was applied to regulate the temperature.

The simulation box was a cuboid of reduced dimensions equal to $L_{x}^{*}$, $L_{y}^{*}$, $L_{z}^{*}$ along the axes *x*, *y*, and *z*, respectively. We assumed that $L_{x}^{*}\;=\;L_{y}^{*}\;=\;L^{*}$ and $L_{z}^{*}\;=\;H^{*}$. $L^{*}$ varied from 100 to 150. Standard periodic boundary conditions in the *x* and *y* directions were assumed. The walls of the box located at $z^{*}\;=\;-0.5H^{*}$ and $z^{*}\;=\;0.5H^{*}$ mimic confining planes. The temperature was kept at $T^{*}\;=\;1$.

Firstly, we carried out simulations for a single hairy particle in a large bulk system. In this case, we studied longer ligands (M = 30). Next, we ran a series of simulations for different wall separations. We started our calculations with the narrowest pore.

We used a bulk configuration and moved the bottom wall as close to the particle as possible. Afterward, we continued the simulation introducing strong interactions with this surface. As a result, the particle fell onto the wall. Then, we slowly pushed the bottom wall away at the position $z^{*}\;=\;-0.5H^{*}$. Next, the top surface was moved to the $z^{*}\;=\;0.5H^{*}$ position. At this stage, we changed segment-wall interactions to repulsive and heated the system to remove all species from the bottom wall. We equilibrated the system again. In this way, the “pre-start” configuration was prepared. In this configuration, the particle was always in the pore center. At the end, we introduced the desired interaction parameters and temperature and started the simulation run. To obtain a “pre-start” configuration for wider pores, we started from the narrowest slit and slowly moved out the confining planes at suitable positions. The described compression/expansion and heating/cooling protocol guaranteed that we obtained the equilibrium structures.

The second series of simulations was performed for a fluid of hairy particles. To shorten the computation time, we assumed that M = 10. The total fluid density was $\rho^{*}\;=\;0.5$. We prepared the initial configurations in the following way. As previously, we started from the systems with repulsive interactions. For each considered wall separation *H*, we took a random configuration of a single particle that was obtained in such a slit. This configuration was replicated in the directions *x* and *y* to obtain 225 particles. This means that we studied 67,725 “atoms”. Next, we compressed the system in the directions *x* and *y* until the desired density $\rho^{*}\;=\;0.5$ was achieved. Then, we introduced the needed parameters of segment-wall interactions and carried out the simulations.

Each system was equilibrated using at least $10^{8}$ time steps until its total energy reached a constant level, at which it fluctuated around the mean value. The production runs were for at least $10^{7}$ time steps. At the time, data were saved after every 100 time steps and used for the evaluation of the needed observables. For each set of the system parameters, we performed several independent simulations.

Examples of the equilibrium configurations are depicted using OVITO [58].

## 4. Conclusions

We carried out a simulation study of model ligand-tethered particles under varying geometric confinement between two inert or attractive walls. We focused on the impact of segment-wall interactions and the slit’s width on the equilibrium configurations of hairy particles. Therefore, we employed a very simple model in which all interactions but the segment-wall ones were assumed to be softly repulsive. We considered particles with mobile ligands, which can slide freely on the surface of the core.

We performed two series of simulations, for single hairy particles between two flat solid surfaces and for slits containing many hairy particles. In the first case, we focused on the impact of the confinement on the shape of individual hairy particle. At the second stage of the work, we investigated the self-assembly of hairy particles under confinement.

In the slits with inert walls, the isolated hairy particles were spheroids, which became increasingly flattened as the pore width rose. These results were consistent with the previous simulations [33]. The most interesting findings were those obtained for attractive walls. In this case, we distinguished two basic conformations of hairy particles: bridges and mounds. The tethered polymers can be connected with both walls and, together with the core, form a bridge between them. Such conformations have not been reported so far.

One can say that the bridges observed in our simulations were similar to flanged spools. We found the following spool-like conformations (symmetrical and asymmetrical): spools (S, S1), hourglass (H, H1), and pillars (P). For the spools, the density of segments has very high and sharp peaks near the walls, and it is relatively high in the central part of the pore. In the case of the symmetric spool, the core is located at the pore center, and its flanges are almost the same, while for asymmetrical spools, the core lies closer to one of the walls and the “spool’s collars” are different. However, for pillars, the segment density is only slightly higher near the walls than in the center. In the case of the hourglasses, the density of segments is very low near the core and gradually increases, reaching the maxima at walls. Most hourglass-like structures are almost symmetrical with the core in the center of the slit. We found, however, asymmetrical hourglasses (H1).

Under certain conditions, the particles fall on one of the walls and resemble mounds. We distinguished three types of structures formed on a single surface: mounds (M), flattened mound (M1), and the mound with a few chains in contact with the other wall (M2). The flattened mound resembles a starfish configuration of adsorbed star polymers, where all chains collapsed onto the surface was observed [31]. The structures formed on a single solid surface were observed experimentally [23] and predicted by computer simulations [26,29].

We showed how the wall separation and the strength of segment-wall interactions affect the parameters describing the shapes of segment clouds, the radius of gyration, its components in the Cartesian coordinates, the prolateness, and the relative shape anisotropy. We concluded that these parameters well characterize general trends of the shape deformation between the walls.

We summarized these structures in the diagram reflecting the impact of the segment-surface interactions and the wall separation on the conformation of the hairy particles with mobile ligands.

We discussed also the behavior of particles with a much smaller number of tethered chains. In this case, we found a very interesting structure, in which the core “jumps” between the walls. Such “nano-oscillators” could be promising building blocks for the production of new materials.

We also modeled the self-assembly of hairy particles under confinement. We considered the morphology of relatively dense systems. We showed how the system morphology depends on interactions with the surfaces and the wall separation. The hairy particles can form different ordered structures, resembling two-dimensional crystals. In the narrowest slits, we found the “levitating layer” of cores located in the center of the system. For wider pores, however, we observed two layers located closer to the walls. We studied the ordering of cores within these layers. If $H^{*}\;=\;7$, the cores are arranged on a hexagonal lattice. However, the symmetry of the layers formed in the slit with $H^{*}\;=\;12$ depends on segment-wall interactions. For repulsive walls, layers of square symmetry are formed. Both layers are shifted with respect to each other. In the case of attractive walls, the cores lie on shifted parallelogram lattices. In the widest slits ($H^{*}\;=\;17$), the cores form two uncorrelated hexagonal layers.

In this work, we concentrated on the effect of confinement between two attractive walls on the behavior of hairy particles. Though we considered the simple model involving only attractive segment-substrate interactions, this approach captures the basic characteristics of hairy particles near surfaces. In real systems, however, other interactions can also play a significant role, for example particle-particle attraction resulting in their aggregation [32] or attractive core-wall interactions. We are currently researching such systems.

In summary, we found that hairy particles confined between two attractive walls form a variety of structures depending on the system parameters assumed. We showed that confinement in attractive slits can be a method for the control of the shape of a single hairy particle. Moreover, our simulations demonstrated that the confinement strongly affects the self-assembly of hairy particles.

This work can be a starting point for the study of the self-assembly of hairy particles in different pores and the adsorption of hairy particles in porous materials. We hope that our results can be helpful in further theoretical research and provide a stimulus for new experiments.

## Data Availability

Not applicable.
